# PreS1 epitope recognition in newborns after vaccination with the third-generation Sci-B-Vac™ vaccine and their relation to the antibody response to hepatitis B surface antigen

**DOI:** 10.1186/1743-422X-6-7

**Published:** 2009-01-20

**Authors:** Ulla B Hellström, Kazimierz Madalinski, Staffan PE Sylvan

**Affiliations:** 1 Department of Communicable Disease Control and Prevention, Uppsala County Council, Sweden; 2 Department of Communicable Disease Control and Prevention, Stockholm County Council, Sweden; 3 National Institute of Public Health – National Institute of Hygiene, Warsaw, Poland; 4 Department of Medical Sciences, Uppsala University, Sweden; 5 The Karolinska Institute, Department of Medicine, Infectious Disease Unit, Karolinska University Hospital, Sweden; 6 Child Health Memorial Institute, Warsaw, Poland

## Abstract

**Background:**

Sci-B-Vac™ is a recombinant, hepatitis B vaccine derived from a mammalian cell line and containing hepatitis B surface antigen (HBsAg) as well as preS1 and preS2 antigens. Few studies have been performed on the antibody responses to preS1 in relation to the antibody to hepatitis B surface antigen (anti-HBs) response during immunisation of healthy children with preS-containing vaccines.

**Results:**

In this study 28 healthy newborns were randomly selected to receive either 2.5 ug or 5.0 ug of the Sci-B-Vac vaccine. Children received three doses of vaccine according to a 0-, 1-, 6-month scheme. Antibodies against the S-protein and three synthetic peptides mimicking three B-cell preS1 epitopes, (21–32 amino acid epitope), (32–47 amino acid epitope) and the C-terminal (amino acid epitope 94–117) were determined at 6 and 9 months. Fourteen (50%) of the 28 newborns had detectable levels of anti-preS1 (21–32) antibodies; 15 (54%) were anti-preS1 (32–47) reactive and 12 (43%) were anti-preS1 (94–117) reactive at 6 or 9 months after initiation of the vaccination. Significantly higher levels of anti-HBs were observed in the sera of patients with detectable anti-preS1 (32–47) reactivity (24 550 ± 7375 IU/L, mean ± SEM) as compared with the non-reactive sera (5991 ± 1530 IU/L, p < 0.05). The anti-HBs levels were significantly lower if none (p < 0.05) or one (p < 0.025) of the preS1 (21–32, 32–47, 94–117) peptides were recognised compared with the anti-HBs levels if two or three peptides were recognised.

**Conclusion:**

Recognition of several preS1 epitopes, and in particular, the epitope contained within the second half of the hepatocyte binding site localised in the hepatitis B surface protein of the third-generation hepatitis B vaccine is accompanied by a more pronounced antibody response to the S-gene-derived protein in healthy newborns.

## Background

Infectious particles of hepatitis B virus (HBV), called Dane particles, consist of viral nucleic acid encapsulated within a core particle enveloped by three distinct virus-coded surface proteins. These three proteins, termed preS1, preS2 and S, are co-terminal at the C-terminus but are different at the N-terminus end regarding the position of the initiation codon of protein translation of the viral genome [1]. It has been demonstrated that the C-terminal part of the preS1 region is essential for viral assembly [2] whereas the N-terminal part is believed to play a major role in mediating virus attachment and entry into hepatocytes [3]. Within the preS1 protein, the 21–47 amino acid epitope was shown to mediate binding to the cell surface of HepG2 cells [4]. Antibodies directed against this epitope were shown to have virus-neutralising activity [5]. A polypeptide covering the N-terminal region 21–47 could inhibit virus-cell interactions, as does the antibody against this fragment [6-8]. Moreover, the hepatitis B surface antigen(HBsAg) preS1 region is highly immunogenic, containing both sequential and conformational epitopes [9] with abundant T- and B-cell epitopes [10-15].

Thus, multiple viral functions of the preS1 region provide a useful target for anti-HBV intervention. Recombinant preS products have been developed as protein vaccines that elicit B- and T-cell immune responses on a broader range of major histocompatibility complex (MHC) haplotypes [16-19]. Comparative immunogenicity studies in mice, rabbits and humans using one such vaccine (BioHep B/Sci-B-Vac™, also known as Hepimmune) have repeatedly confirmed the excellent immunogenicity measured as antibody to hepatitis B surface antigen (anti-HBs) production and safety of this third-generation HBV vaccine [20]. However, only a few studies have been performed on the antibody responses to preS1 in relation to the anti-HBs response during immunisation of healthy children with preS-containing vaccines.

Recently, we demonstrated that recognition of the preS epitopes contained in the third-generation preS1/preS2/S vaccine (Sci-B-Vac™, BioHepB) is accompanied by a more rapid onset and pronounced antibody response to the S-gene-derived protein in healthy children and newborns [21,22]. To further analyse the specificity and significance of the antibody responses toward the two linear preS1 sequences that have been shown to represent human B cell epitopes within the hepatocyte binding site comprising amino acids preS1 (21–47) [[4,6,7], reviewed in [23]], as well as the C-terminal part comprising amino acids (94–117) [24,25], we measured the specific antibody response in healthy newborns after immunisation with the Sci-B-Vac™ vaccine. Furthermore, we evaluated whether induction of antibodies toward the three preS1 epitopes (21–32, 32–47 and 94–117) is associated with an enhanced response to HBsAg. We found that recognition of several preS1 epitopes, and in particular, the epitope contained within the second half of the hepatocyte binding site localised in the hepatitis B surface protein of the third-generation hepatitis B vaccine is accompanied by a more pronounced antibody response to the S-gene-derived protein in healthy newborns.

## Methods

### Vaccine

Immunisation of newborns was performed with the recombinant Chinese hamster ovary (rCHO) cell-derived Sci-B-Vac™ vaccine, (SciGen Ltd, Singapore, earlier referred to as Bio-Hep-B™ vaccine, Bio-Technology General Corporation) [19]. The vaccine was purified from the culture media of CHO cells transfected with the nucleotide sequences coding for all three surface antigens (i.e. preS1, preS2 and S). The Sci-B-Vac™ vaccine of the HBsAg subtype *adw*_2 _and genotype A is > 99% pure and the proteins of the vaccine are absorbed on alum phosphate (0.5 mg/ml); the preservative agent is Thiomerosal (50 ug/ml). The vaccine was stored and transported at 2–8°C. The same batch of vaccine was used throughout the study. The vaccinees received three doses of vaccine according to a 0-, 1-, and 6-month schedule [21].

### Study design

Twenty-eight healthy newborns were qualified for vaccination. Fifteen received 2.5 ug doses and 13 received 5.0 ug doses of vaccine. The selection of infants was random. The mothers were negative for hepatitis B markers (HBsAg, antibody to hepatitis B core antigen (anti-HBc) and anti-HBs). The cord blood samples were also negative for these markers. The transaminase levels (ALT) in mother and cord blood samples were within the normal range. The newborns were evaluated within the first 5 min of life as having at least 7 points on the Apgar scale. The newborns' body weight was > 2500 g. Newborns from drug-addicted or alcoholic parents were excluded from the study. Anti-HBs and anti-preS1 antibodies were evaluated at 6 (T6, i.e. 5 months after the second injection) and 9 months (T9, i.e. 3 months after the third injection) after vaccination with the Sci-B-Vac™ vaccine.

In accordance with the Helsinki declaration, parents of all children had signed an informed consent form for participation in the study, The parents were instructed on how the vaccine was tested for safety and immunogenicity in adults and how to observe local signs and general symptoms associated with the vaccine administration. The frequencies of all signs and symptoms (i.e. vaccine reactions) that the parents observed were reported in their diary cards. These signs might include appetite loss, diarrhoea, fever, irritability, sleeplessness and vomiting, as well as the local signs of pain and redness or swelling at the injection site. The study was approved by the Ethics Committee of the Child health Memorial Institute [21].

### Antibody detection

Anti-HBs antibodies were measured using a microparticle enzyme immunoassay in an IMX apparatus (Abbott, Chicago, ILL., USA) and expressed as IU/L. Control tests (hepatitis B, anti-HBs from Labquality, Helsinki, Finland) were used throughout the study. Antibodies to preS1 were assessed by enzyme-linked immunosorbent assay (ELISA). Microtitreplates (Immunolon 2, no.011-010-3455, Dynatech, Chantilly, VA, USA) were coated with preS1 (21–32), (32–47), (94–117) (*adw*_2_) peptídes (purchased from Sigma Genosys, Cambridge, UK) at a concentration of 2 ug/ml in 0.05 M sodium carbonate buffer (pH 9.6) at 4°C overnight.

Patient and pooled control sera, diluted 1/125 in 0.05 M phosphate-buffered saline (PBS)-Tween-1% foetal bovine serum (FBS), were incubated on the plates at 4°C overnight. The plates were washed and incubated with alkaline phosphatase (ALP)-conjugated goat anti-human gamma chains (A-3187, Sigma Chemical Company, St. Louis, MO, USA) and diluted 1:1000 in PBS-Tween-1% FBS at 4°C overnight. After incubation (5–30 min) with p-nitrophenyl phosphate in diethanolamine HCl, the optical density (OD) at 405 nm was measured in a Titertek Multiskan Plus Photometer (Flow Laboratories, Edinburgh, Scotland). Sera from 20 hepatitis B-susceptible healthy blood donors, routinely tested and lacking serum markers for hepatitis A-E, were pooled and used as controls to obtain the normal (N) value. Positive antibody reactivity was defined as a sample (S) over the N value (S/N ≥ 2.5) equal to the mean OD value plus 4 SD of the control sample.

### Specificity tests

**Figure 1:**
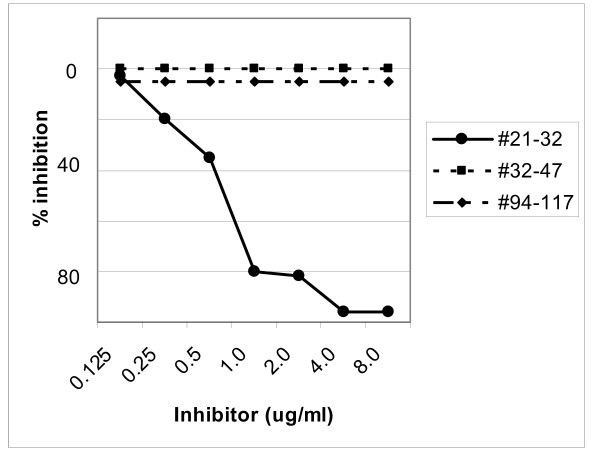
**Inhibition of IgG anti-preS1(21–32) reactivity**. A diluted serum sample from an anti-preS1 (21–32) reactive patient was preincubated with different concentrations (0.125–8.0 ug/mL) of the synthetic peptide analogue corresponding to the (black circle) 21–32, (black square) 32–47 or (black rhomboid) 94–117 amino acid sequences of preS1 before assaying in the preS1 (21–32) ELISA.

**Figure 2:**
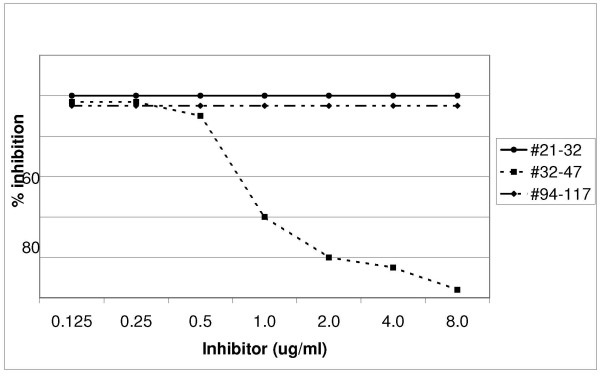
**Inhibition of IgG anti-preS1 (32–47) reactivity**. A diluted serum sample from an anti-preS1 (32–47) reactive patient was preincubated with different concentrations (0.125–8.0 ug/mL) of the synthetic peptide analogue corresponding to the (black circle) 21–32, (black square) 32–47 or (black rhomboid) 94–117 amino acid sequences of preS1 before assaying in the preS1 (32–47) ELISA.

Equal volumes of diluted serum samples from anti-preS1 (21–32) or (32–47) reactive individuals and inhibitors (0.125–8.0 ug/mL) were incubated for 4 h at 4°C before addition to the ELISA plates and further analysed as described above. The synthetic peptide analogues preS1 *adw*_2 _(21–32), (32–47) or (94–117) were used as inhibitors. The relevant preS1 peptide inhibited the preS1 reactivity to 100% whereas irrelevant preS1 peptides did not (Figures 1 and 2). Anti-preS1 (94–117) specificity has been documented earlier [25].

### Statistical analysis

The non-parametric Mann-Whitney U test was employed to compare data between groups of newborns vaccinated with different doses of the Sci-B-Vac™ vaccine.

## Results

The presence of IgG antibodies with specificity for the synthetic peptide analogues corresponding to the preS1 (21–32), (32–47) and (94–117) regions of the HBV was studied using peptide-based ELISA at a 1/125 dilution of sera from 28 newborns after complete immunisation with the Sci-B-Vac™ vaccine. Fourteen (50%) of the 28 newborns had detectable levels of anti-preS1 (21–32) antibodies at 6 or 9 months after initiation of the vaccination. Fifteen (54%) and 12 (43%) newborns were anti-preS1 (32–47) and (94–117) reactive, respectively. No significant difference in response to the preS1 epitopes was noted in newborns immunised with the 2.5 ug or the 5.0 ug vaccine doses.

The mean levels of anti-HBs were significantly higher in the anti-preS1 (32–47) reactive sera (24 550 ± 7375 IU/L, mean ± SEM) as compared with the non-reactive sera (5991 ± 1530 IU/L, p < 0.05) (Table 1). In contrast, no significant difference was noted between anti-preS1 (21–32) or (94–117) reactive group compared with their respective non-reactive groups.

**Table 1:** Anti-HBs titres in preS1 (21–32, 32–47 and 94–117) reactive sera compared with non-reactive sera from newborns vaccinated with Bio-HepB™ vaccine

	Reactive	Non-reactive	Probability
Anti-preS1 (21–32)	n = 14	n = 14	
Anti-HBs IU/L	15 841 ± 4 023	16 026 ± 7 859	ns
			
Anti-preS1 (32–47)	n = 15	n = 13	
Anti-HBs IU/L	24 550 ± 7 375	5 991 ± 1 530	p < 0.05
			
Anti-preS1 (94–117)	n = 12	n = 16	
Anti-HBs IU/L	19 464 ± 5 280	13 286 ± 6 533	ns

ns = not significant

Anti-HBs (mean ± SEM)

Table 2 demonstrates that the anti-HBs levels were significantly lower if none (p < 0.05) or one (p < 0.025) of the preS1 (21–32, 32–47, 94–117) peptides were recognised in comparison with the anti-HBs levels if two or three peptides were recognised.

**Table 2:** Anti-HBs levels in vaccinated newborns with different anti-preS1 peptide (21–32, 32–47, 94–117) reactivities at time T9

Reactivity with	Number	Anti-HBs (IU/L)	Probability*
3 preS1 peptides	n = 7	23 688 ± 6 183 a/	
2 preS1 peptides	n = 4	22 890 ± 10 670 b/	ns
1 preS1 peptide	n = 12	11 919 ± 8 669 c/	p < 0.025
0 preS1 peptide	n = 5	2 065 ± 1 105 d/	p < 0.05

*the a/ compared with the b/, c/ or d/ groups of vaccinated newborns (Mann-Whitney test)

ns = not significant

T9 = three months after the third injection

Anti-HBs (mean ± SEM)

## Discussion

A large number of studies have suggested a direct involvement of the preS1 domain of the hepatitis B virus large envelope protein (L-HBsAg) (in particular amino acids 21 to 47) in a virus attachment to hepatocytes [[4,6,7], reviewed in [23]]. Recently, it was demonstrated that a mutant L-HBsAg bearing a deletion in the 26–30 amino acid sequence of the preS1 receptor binding site was non-infectious and hence deficient in viral entry [3], further supporting the importance of this domain as an infectivity determinant in hepatitis B. The aim in this study was to identify preS1 antibodies reacting with two human antibody binding sites, p (21–32), containing an infectivity determinant and p (32–47), including a previously defined HBV-neutralisation epitope comprising amino acids 37–45 of preS1 that have been identified within this sequence [26] as well as preS1 antibodies reacting with the C-terminal part (94–117) in sera from newborns completely immunised with the third-generation recombinant vaccine (Sci-B-Vac™).

We found that immunised newborns exhibited anti-preS1 (21–32) and anti-preS1 (32–47) and anti-preS1 (94–117) antibody reactivity in 50% (14/28), 54% (15/28) and 43% (12/28), respectively. The prevalence of vaccine-induced anti-peptide antibodies in this study is identical to the prevalence of antibodies reacting with p (21–32), p (32–47) and p (94–117) in sera obtained during convalescence from natural HBV infection [24]. The third-generation vaccine used in this study, Sci-B-Vac™, was developed using mammalian cells, which provide envelope proteins similar to those isolated from infected patients and used for preparation of some of the first-generation HBV vaccines [20]. This similarity could explain the identical immunogenicity and specificity induced by natural infection or complete vaccination with a preS1-, preS2- and S-containing hepatitis B vaccine.

Five of 28 (18%) newborns lacked detectable levels of anti-preS1 (21–32), anti-preS1 (32–47) or anti-preS1 (94–117) antibodies. One explanation for this observation could be that the preS1 (21–32; 32–47 and 94–117) non-reactive patients have conformation dependent anti-preS1 antibodies in the circulation that are not reactive with the linear peptide analogues of preS1 (21–32, 32–47 and 94–117) used in this study. In most cases synthetic peptides are considered unsuitable for studying the discontinuously conformational epitopes because most peptide-specific antibodies are continuous sequence-specific [27]. The three-dimensional structure of the HBV surface protein, including preS1, has not yet been determined, though Lian et al [28] recently demonstrated that amino acids 31–36 were pivotal for the folding and conformational stability of the preS protein.

We demonstrated that those newborns that developed antibody reactivity against two or three B-cell epitopes had a significantly stronger anti-HBs response than those individuals that showed a narrower preS1 response. In congenic mice inclusion of preS regions elicits a broader spectrum of protective antibodies that augment the anti-HBs response and circumvent non-responsiveness to the S protein [26].

The preS1 region in humans has been shown to be a particularly efficient immunogen at the T-cell level. A dominant T-cell recognition site was identified in the N-terminal residues 21–28 (serotype *adw*) of the preS1 sequence [29,30] and in vitro binding studies have defined preS1 (25–33) as a promising helper T-cell epitope [31]. Further studies will show whether preS1 T-cell epitopes contained within the third-generation hepatitis B vaccine can mediate helper T-cell functions on the human antibody response to the particulate HBsAg similarly to what has been shown in the mouse system.

## List of abbreviations

HBV: hepatitis B virus; HBsAg: hepatitis B surface antigen; Anti-HBc: antibody to hepatitis B core antigen; Anti-HBs: antibody to hepatitis B surface antigen; ALT: alanine aminotransferase; OD: optical density; N: normal; S: sample.

## Financial competing interest

This study has been partly financed by SciGen Ltd, Singapore

## Authors' contributions

UBH, KM and SPES participated in developing the study concept, research design, and analytical approach; interpreting the data; and developing the manuscript (writing or giving substantive input).
